# Over-the-Counter Biosensors: Past, Present, and Future

**DOI:** 10.3390/s8095535

**Published:** 2008-09-06

**Authors:** Thomas Ming-Hung Lee

**Affiliations:** Department of Health Technology and Informatics, The Hong Kong Polytechnic University, Hung Hom, Kowloon, Hong Kong; E-mail: htmhlee@polyu.edu.hk; Tel.: (852)-2766-4931; Fax: (852)-2362-4365

**Keywords:** Home-use biosensors, glucose sensors, metabolite sensors, protein sensors, DNA sensors

## Abstract

The demand for specific, low cost, rapid, sensitive and easy detection of biomolecules is huge. A well-known example is the glucose meters used by diabetics to monitor their blood glucose levels. Nowadays, a vast majority of the glucose meters are based on electrochemical biosensor technology. The inherent small size and simple construction of the electrochemical transducer and instrument are ideally suited for point-of-care biosensing. Besides glucose, a wide variety of electrochemical biosensors have been developed for the measurements of some other key metabolites, proteins, and nucleic acids. Nevertheless, unlike the glucose meters, limited success has been achieved for the commercialization of the protein and nucleic acid biosensors. In this review article, key technologies on the electrochemical detection of key metabolites, proteins, and DNAs are discussed in detail, with particular emphasis on those that are compatible to home-use setting. Moreover, emerging technologies of lab-on-a-chip microdevices and nanosensors (i.e., silicon and carbon nanotube field-effect sensors) offer opportunities for the construction of new generation biosensors with much better performances. Together with the continuous innovations in the basic components of biosensors (i.e., transducers, biorecognition molecules, immobilization and signal transduction schemes), consumers could soon buy different kinds of biosensing devices in the pharmacy stores.

## 1. Introduction

The groundbreaking work on enzyme electrodes by Clark and Lyons in 1962 [1] marked the beginning of the field of biosensors. Generally speaking, a biosensor is a device that couples a biorecognition element with a transducer, and converts the recognition event into a useful analytical signal (preferably an electrical signal). Over the past few decades, numerous biosensors have been developed for the detection of ions, small molecules, proteins, deoxyribonucleic acids (DNAs), cells and many others. They have been used in a wide range of applications from medical diagnostics [2], food quality assurance [3], environmental monitoring [4], industrial process control [5, 6] to biological warfare agent detection [7]. Not surprisingly, great efforts have been devoted to their commercialization. At present, the global market for biosensors is about $7 billion, with home-use health monitoring devices (e.g., glucose biosensors and pregnancy test strips) being dominant. These devices provide accurate results in no time and at low cost.

So far, the transduction principles employed in these home-use biosensors are mainly based on electrochemistry or reflectance/absorption technique (quantitative measurement for color-forming chemistry) due to their inherent simple instrumentation and small size. For glucose biosensors, the majority of current devices are of the electrochemical type attributed to better analytical performance as well as easier instrument maintenance. In fact, significant advancements have also been made for electrochemical/electrical detection of proteins and DNAs. It is very likely that these biosensors will soon be available on the market for widespread use. In this review article, the historical development, current research activities, as well as potential challenges in electrochemical/electrical detection of key metabolites, proteins, and DNAs are discussed. Particular attention is given to the growing importance of microelectromechanical systems (MEMS) and nanotechnology in biosensing applications.

## 2. Metabolite Sensors

The concentrations of key metabolites in our body are usually maintained within their physiological ranges. Deviation from the normal range is indicative of certain illnesses. A well-known example is diabetes, which is characterized by elevated blood glucose concentration as a result of no/insufficient insulin production in the pancreas or insulin resistance. Diabetics must strive to achieve good glycemic control in order to avoid complications such as blindness, heart and kidney diseases. One prerequisite for such tight control is accurate and frequent monitoring of the blood glucose level that provides useful information to guide a treatment plan (i.e., dosage of insulin or diabetes pill). Tens of pocket-size glucose meters are now available to meet the needs of the diabetics.

### 2.1. Basic Principles of Electrochemical Glucose Biosensors

A number of excellent reviews on glucose biosensors have been published [8-12]. Herein, key technologies are described, and the current market situation as well as future prospects is emphasized. The first glucose biosensor illustrated by Clark and Lyons comprised an oxygen electrode, an inner oxygen semipermeable membrane, a thin layer of glucose oxidase and an outer dialysis membrane [1]. The outer membrane keeps the enzyme in close proximity to the electrode surface and controls the diffusion of glucose as well as oxygen. Meanwhile, the inner membrane allows oxygen to pass through and blocks some electroactive interferents from reaching the electrode. Glucose oxidase (GOD) catalyzes the oxidation of glucose to gluconolactone, and the redox cofactor (i.e., flavin adenine dinucleotide, FAD) of GOD is reduced to FADH_2_:

(1) $$\text{glucose}+\text{GOD}(\text{FAD})\to \text{gluconolactone}+\text{GOD}({\text{FADH}}_{2})$$

The cofactor is regenerated by reaction with oxygen, leading to the formation of hydrogen peroxide. In addition, gluconolactone is hydrolyzed to gluconic acid:

(2) $$\text{GOD}({\text{FADH}}_{2})+{\text{O}}_{2}\to \text{GOD}(\text{FAD})+{\text{H}}_{2}{\text{O}}_{2}$$

(3) $$\text{gluconolactone}+{\text{H}}_{2}\text{O}\to \text{gluconic acid}$$

The amperometric signal from the reduction of oxygen is used to determine the concentration of glucose in the sample. As oxygen is consumed, hence the current signal decreases with increasing glucose concentration. One major drawback of this approach is the fluctuation of the background oxygen level, thus adversely affecting the sensor's accuracy. This issue was addressed by Updick and Hicks using a dual oxygen electrode [13], one with active enzyme on its surface while the other one with heat inactivated enzyme, of which the differential current output eliminates the effect from changing background oxygen concentration. Nevertheless, it should be noted that the construction of the dual electrode is more complicated than the single electrode.

Besides oxygen, the hydrogen peroxide produced can be electrochemically oxidized to determine the glucose concentration [14]. When a platinum electrode is used, the potential required is about +0.7 V versus Ag/AgCl reference electrode. Such high positive potential can also oxidize some other compounds such as ascorbic acid and paracetamol. Analogous to the oxygen measurement, these interferences can be minimized by the two-membrane configuration. The first successful commercial glucose biosensor from Yellow Springs Instrument in 1975 was based on the hydrogen peroxide approach, with a cellulose acetate inner membrane and a polycarbonate outer membrane. This analyzer was almost exclusively used in clinical laboratories because of its high cost.

It took twelve more years for glucose biosensors to go from clinical to home use. Two breakthroughs led to the realization of a pen-size glucose biosensor (i.e., ExacTech marketed by MediSense, now owned by Abbott), namely redox mediator and screen printing technologies. The oxygen-dependent glucose biosensors (i.e., both oxygen and hydrogen peroxide approaches, classified as the first generation type) are difficult to manufacture in large scale due to the membranes involved. In the 1970s and 1980s, ferricyanide [15] and ferricinium [16] ions have been demonstrated to be efficient electron acceptors for glucose oxidase. Of particular significance is the lower detection potential for these redox species (about +0.3 V versus Ag/AgCl reference electrode), at which the oxidization of common interferences are suppressed and thus the membranes can be omitted. This redox mediator-based approach is termed as the second generation glucose biosensors. Another breakthrough contributes to the development of disposable test strips that are much simpler and cheaper to manufacture than the platinum rod/wire electrodes. The sensing and reference electrodes, in the form of thick inks (e.g., carbon and metal pastes), are screen-printed onto a ceramic or plastic substrate.

The electron transfer from the redox center (FADH_2_) to the electrode of the first and second generation glucose biosensors relies on soluble electron acceptors in that the redox center is embedded within the enzyme's glycoprotein body. One major disadvantage associated with the redox mediators is their high toxicity. Leakage of these small molecules from the electrode surfaces is unavoidable, so the second generation glucose biosensors are not suitable for *in vivo* conditions. In view of this, various strategies have been employed to electrically wire the redox enzymes to the electrodes, regarded as the third generation glucose biosensors. Heller's group showed that an average of 12 ferrocenecarboxylic acid molecules covalently attached to each glucose oxidase molecule promoted electron transfer at practical rates [17]. Apart from chemical modification of the enzyme, the same group established the electrical wiring by immobilizing the enzyme within a redox hydrogel formed by poly(1-vinylimidazole) complexed with Os(4,4′-dimethylbpy)_2_Cl cross-linked with poly(ethylene glycol) diglycidyl ether [18]. Yet another wiring approach was devised by Willner's group based on the reconstitution of apo-glucose oxidase with a monolayer of FAD immobilized onto the electrode surface via different relaying units [19, 20].

### 2.2. Types of Glucose Meters

Until now, the operation of most commercial glucose meters is not much different from that of the ExacTech meter. A test strip is first inserted to the meter, then a small drop of blood is obtained from fingertip with a lancing device and is applied to the test strip, finally the result is displayed. These *in vitro* measurements provide only discrete data and are invasive in nature.

Another type of glucose biosensors is for continuous glucose monitoring. This is particularly useful in providing real-time feedback control to insulin pump. The earliest *in vivo* glucose biosensor was reported by Shichiri *et al.* [21], the design of which was fundamentally the same as the glucose biosensors made by Clark and Lyons [1] as well as Updick and Hicks [13], except the diameter of the sensor body was much smaller (0.4 mm). This needle format facilitates the insertion of the sensor in subcutaneous tissue. Crucial parameters of this needle-type sensor include biocompatibility, calibration, long-term stability, specificity, and linearity. Numerous studies have been carried out to improve the performance of these needle-type glucose sensors [22-31].

Continuous subcutaneous glucose monitoring can also be achieved without direct contact between the interstitial fluid and transducer using microdialysis technique [32, 33]. A semipermeable dialysis fibre is inserted subcutaneously through the skin with the assistance of an 18-guage needle. Glucose-free physiological saline solution is then pumped through the fibre to extract glucose molecules from the interstitial fluid by diffusion. The dialysate is transported to a sensing unit outside the body and the measurement is made by detecting the hydrogen peroxide generated from the enzymatic reaction.

One expected complaint from the needle- and microdialysis-type glucose sensors is the pain involved in the invasive implantation procedure. Several minimally invasive techniques have thus been developed for the extraction of glucose through the skin. The best known one is reverse iontophoresis [34, 35]. Basically, when an electrical current is applied to the skin surface, the interstitial fluid crosses the stratum corneum barrier as a result of electroosmotic flow. The extracted glucose molecules are collected by a glucose oxidase containing hydrogel disk and the hydrogen peroxide generated is detected by a screen-print electrode in contact with the hydrogel.

### 2.3. Current Glucose Meters Market Situation

According to the International Diabetes Federation, there are currently 246 million diabetics worldwide and the number is expected to reach 380 million by 2025. With this, the demand for glucose biosensors is huge and the business is very profitable. This market, in the past 10 years, has been led by four companies: Abbott, Bayer, LifeScan, and Roche. The fingerprick-type glucose biosensors have enjoyed the greatest commercial success. While many different meters are now available on the market, in practice their performances do not differ much from one another. A brief summary of the key features of four representative meters is given in Table 1.

The fingerprick-type glucose biosensors have also been extended to other key metabolites such as urea, creatinine, lactate (technical specifications of some commercial devices are given in Table 2), uric acid, cholesterol, and ketone [36]. It should be pointed out that a single meter can be used for the detection of multiple analytes, e.g., Abbott's Precision Xtra Advanced Diabetes Management System measures both blood glucose and b-ketone with two types of test strips fitted to the same instrument. Another more sophisticated device, i-STAT handheld blood analyzer (also under Abbott's umbrella after a $392 million acquisition in 2003), allows simultaneous measurements of multiple analytes in a disposable cartridge format. This device is mainly used by clinicians as the interpretation of results is not straightforward.

The first commercial needle-type glucose biosensor was marketed by Minimed (Continuous Glucose Monitoring System, CGMS). The CGMS does not display the measured glucose concentration, but stores the results (data interval of 5 min) in a 3-day operation cycle. An updated system, the Guardian Real-Time System, offers real-time display of the results. More importantly, this continuous sensor can work with an insulin pump (i.e., Minimed Paradigm Real-Time System) to provide more effective therapy. Abbott very recently released a new needle-type continuous glucose monitoring system called FreeStyle Navigator that can be continuously worn on body for up to 5 days, while Menarini's microdialysis-based GlucoDay S can operate for 2 days. Yet another commercial continuous glucose monitor is the GlucoWatch by Cygnus (now owned by Animas, a Johnson & Johnson company), which measures glucose 3 times per hour for 12 hours after a 3-hour warm-up period. However, the company has ceased to sell this product since August 2007, possibly attributable to the warm-up procedure and chance of getting skin irritation.

### 2.4. Future Prospects of Metabolite Biosensors

**Figure 1.**
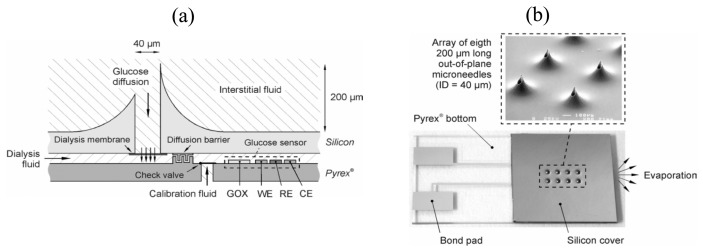
(a) Schematic diagram and (b) photographs of a microneedle-based glucose monitor. From Zimmermann *et al.*, *Transducer '03, The 12^th^ International Conference on Solid State Sensors, Actuators and Microsystems*, pp. 99-102. Reprinted with permission from IEEE (© 2003 IEEE).

Advancements in the sensing chemistry, signal transduction mechanism, sensor fabrication methods, as well as data management, have resulted in the continual introduction of better performing metabolite biosensors. In the foreseeable future, MEMS and nanotechnology are going to make significant impact on next generation devices. MEMS refers to systems or devices that contain electrical (e.g., electrode) and/or mechanical (e.g., pump and valve) components with critical dimensions of 1 to 100 micrometers. In fact, microfabrication technologies have already been utilized in the construction of certain commercial biosensors discussed above. The i-STAT analyzer has an array of microelectrodes and the immobilization of the biorecognition elements (e.g., glucose oxidase) onto the electrode is achieved by photolithography. In addition to the enhanced multiplexing capability, MEMS holds great promise for minimally invasive metabolite sensing. Liepmann and co-workers developed hollow microneedles in a silicon substrate for pain-free extraction of glucose molecules from the interstitial fluid (Figure 1a) [37]. The inner diameter and length of these microneedles was 40 and 200 micrometers, respectively, which were long enough to penetrate the stratum corneum but too short to reach the nerve fibres. The silicon substrate was bonded to a glass substrate with an integrated glucose sensing unit (Figure 1b). When the microneedles were pressed onto the skin, glucose diffused into the dialysis fluid through a polysilicon dialysis membrane, where protein molecules were prevented from reaching the sensing unit so as to improve its long-term stability. Glucose oxidase was immobilized upstream of the sensing unit and hydrogen peroxide generated was measured amperometrically. The driving force for fluid flow was by capillary action and evaporation.

**Figure 2.**
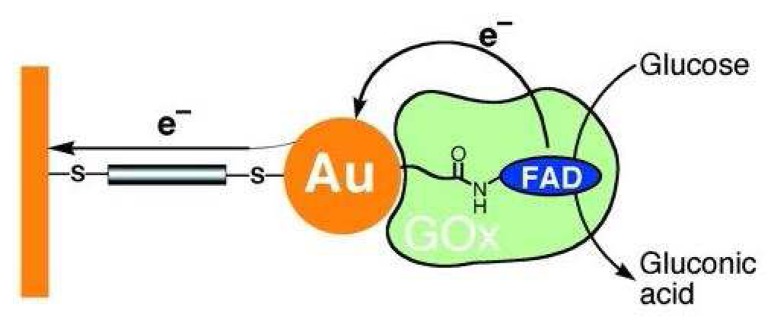
A schematic representation of a gold nanoparticle-reconstituted glucose oxidase electrode. From Xiao *et al. Science* **2003**, *299*, 1877-1881. Reprinted with permission from AAAS.

Nanotechnology is the understanding and control of matter at dimensions of 1 to 100 nanometers, where unique phenomena enable novel applications. Glucose biosensors, in no doubt, can also benefit from this emerging technology. For example, Willner et al. reported that the reconstitution of apo-glucose oxidase with FAD linked to 1.4-nanometer gold nanocrystal, followed by assembly on a gold electrode functionalized with a 1,4-dimercaptoxylene monolayer, yielded a superior bioelectrocatalytic system (Figure 2) [38]. The electron transfer rate of this gold nanoparticle-based system was 7 times higher that than with oxygen as electron acceptor.

## 3. Protein Sensors

Proteins play important roles in many biological processes such as metabolism (enzymes), cell signaling (receptors), and immune response (antibodies). The presence or absence or the amount of certain proteins within our body have proved to be useful biomarkers of our health status. Some well-known examples include human chorionic gonadotropin (hCG) for pregnancy, prostate specific antigen (PSA) for prostate cancer, cardiac troponin I (cTnI) for myocardial infarction, and many others. In clinical laboratories, the most common protein detection method is enzyme-linked immunosorbent assay (ELISA), which makes use of one or more antibodies to recognize a target antigen specifically. The enzyme labels convert the recognition event into colorimetric, chemiluminescence, or electrochemical signal. Depending upon the required sensitivity and specificity as well as the antigen itself, several immunoassay formats can be employed including homogeneous/heterogeneous, direct/indirect, and sandwich/competitive. In general, a heterogeneous, direct, and sandwich ELISA would result in the highest sensitivity and specificity. Typically, a monoclonal antibody (capture antibody) is immobilized onto a 96-well microtiter plate, followed by a blocking step to minimize subsequent nonspecific binding. Then, a sample is introduced and an antibody–antigen complex is formed when the sample contains the target antigen. After that, an enzyme-conjugated secondary antibody that binds to a different epitope than the capture antibody is applied. Finally, an enzyme substrate is added and the reaction product is measured. A thorough washing is needed between each of the above steps in order to remove the excess reagent and nonspecifically bound substances. The microtiter plate format is suitable for high-throughput analysis, but not for home testing. It should be pointed out that ELISA on microtiter plate is considered as bioassay but not biosensor because the recognition element is not in close contact with the transducer.

Since the pioneering work by Janata in 1975, electrochemical immunosensors, with an antibody or antigen in close contact with an electrode, have received tremendous attention. Different transduction strategies have been developed including potentiometry [39, 40], amperometry [41-47], capacitance [48, 49], impedance [50, 51], and field-effect transistor (FET) [52, 53]. Though the size of these sensors is small, almost all of them have to be operated by trained personnel due to the complex procedure involved. The amperometric immunosensors enjoy the signal amplification offered by the enzyme labels and hence can detect trace amounts of target analytes (sub ng/mL levels). Analogous to the microtiter plate format, the main disadvantage is the cumbersome washing steps. In an attempt to automate the entire assay procedure, a number of studies have coupled electrochemical immunosensor to flow injection system [54, 55]. However, the flow injection system is again not suitable for home testing.

Various separation-free strategies have been developed so as to simplify the amperometric assay procedure. A novel method was reported by Duan and Meyerhoff that enabled preferential measurement of surface-bound enzyme-labeled antibody than the unbound ones in the bulk solution [56]. A microporous nylon membrane with a thin-film of gold sputtered on one side was mounted between two chambers of a diffusion cell. Anti-hCG monoclonal capture antibody was immobilized covalently onto the gold layer via a self-assembled monolayer of thioctic acid. The sample together with an alkaline phosphatase-anti-hCG antibody conjugate was added to the gold side of the diffusion cell. After a 30-min incubation, the enzyme's substrate (i.e., 4-aminophenyl phosphate) was added to the other side of the diffusion cell. In the presence of hCG, a sandwich was formed and the alkaline phosphatase was brought close to the gold surface, which served as the working electrode. The substrate diffused through the membrane and reacted with the enzyme bound to the gold surface first. The reaction product (aminophenol) was detected immediately by oxidation at the gold electrode. Another separation-free strategy was based on enzyme channeling [57, 58]. It had a dual enzyme design, with one enzyme covalently attached to the electrode and the other one conjugated to a secondary antibody in a sandwich assay [57] or to a standard antigen in a competitive assay [58]. When the latter one was brought close to the electrode surface through antibody–antigen interaction, the reaction product of one enzyme served as the reactant for the other one. With this, the background signal from the unbound enzyme label in the solution was negligible.

**Figure 3.**
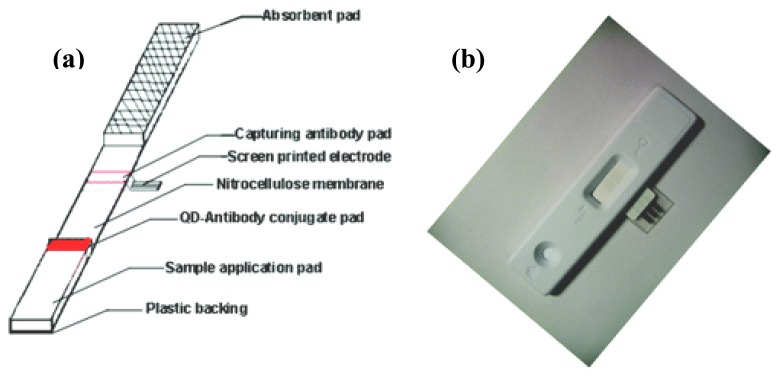
(a) Schematic illustration and (b) photograph of the disposable electrochemical immunosensor diagnosis device. From Liu *et al.*, *Anal. Chem.* **2007**, *79*, 7644-7653. Reprinted with permission from ACS.

So far, the most successful home-use protein detection device is for pregnancy test. The assay is based on lateral-flow immunochromatographic technique [59]. The device is typically constructed with a nitrocellulose membrane with a sample addition pad and an absorbent pad at the two ends. A conjugate pad, which embeds monoclonal anti-hCG antibody conjugated with dye label (e.g., gold nanoparticle and dye-doped polystyrene micro/nanosphere), is sandwiched between the sample addition pad and the nitrocellulose membrane. Close to the absorbent pad end of the nitrocellulose membrane contains one test line and one control line with antibodies against hCG and the monoclonal antibody immobilized on it, respectively. When a urine sample is applied to the sample addition pad, it is transported toward the absorbent pad by capillary action. In the presence of hCG, the dye label is captured in both the test and control lines. Otherwise, only the control line is colored. Strictly speaking, this device cannot be classified as a biosensor as the result is read by naked eyes and no transducer is involved. In fact, it is possible to obtain a semi-quantitative result by incorporating a reflectance-based reader (e.g., Clearblue's Easy Digital Pregnancy Test). In recent years, efforts have been made in integrating the lateral-flow immunochromatographic technique with electrochemical detection system. McNeil et al. demonstrated an impedimetric measurement of the antibody–antigen interaction occurred at the test line [60, 61]. Instead of a dye label, the mobile monoclonal antibody was labeled with urease. Immediately after the capture of the monoclonal antibody–urease conjugate at the test line, a urea solution was allowed to flow through the test line to wash away the unbound materials, and was, in the meantime, hydrolyzed by the urease to effect a localized increase of pH. A pH-sensitive polymer-coated electrode was positioned directly over the test line so that the pH change induced a breakdown of the polymer layer and thus a measurable change in the capacitance of the electrode. Besides enzyme label, Lin et al. reported a highly sensitive assay utilizing quantum dot label (CdS@ZnS) [62]. Regarding the electrochemical detection scheme, the quantum dot label was first dissolved by a simple acidic treatment and the amount of cadmium ions was determined by stripping voltammetric measurement with a disposable screen-printed electrode placed underneath the nitrocellulose membrane at the test line (Figure 3). Both impedimetric and stripping voltammetric methods allow quantitative measurements to be made. Also, they are usually much more sensitive than reflectance-based approach.

**Figure 4.**
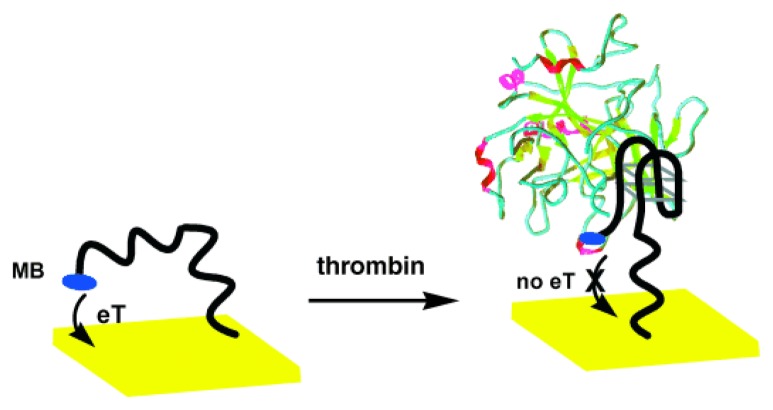
A schematic of the label-free electrochemical aptamer-based protein sensor. From Yi Xiao *et al.*, *Angew. Chem. Int. Ed.* **2005**, *44*, 5456-5459. Copyright Wiley-VCH Verlag GmbH & Co. KGaA. Reprinted with permission.

Aptamers, which are synthetic nucleic acid molecules that bind to non-nucleic acid targets (e.g., small molecules, proteins, and cells) with high specificity and affinity, have emerged as a promising protein recognition element. They are obtained through an *in vitro* selection process known as systematic evolution of ligands by exponential enrichment (SELEX). When compared to antibodies, aptamers are much easier to synthesize and have higher stability. Over the past few years, a number of aptamer-based electrochemical protein detection schemes have been reported [63-77]. In particular, the conformational change of aptamers upon target binding offers unique opportunities in achieving label-free detection. Plaxco and co-workers immobilized a thrombin-binding aptamer to a gold electrode with its thiol group at the 5′ end [67]. The 3′ end of the aptamer had a covalently attached methylene blue redox marker. As shown in Figure 4, in the absence of thrombin, the aptamer assumed an unfolded state, thereby facilitating electron transfer (eT) between the redox marker and the electrode, whereas in the presence of thrombin, the aptamer became folded and eT was reduced (signal-off configuration). Other favorable features of this aptasensor included the capability of separation-free and real-time measurements. By manipulating the sequence of the non-thrombin binding region of the aptamer, O'Sullivan and co-workers turned the operation of the previous aptasensor to a signal-on configuration [68]. The signal-off architecture was based on a 32-mer sequence (with a spacer of 15 bases at the immobilization end) while the signal-on architecture had no spacer.

**Figure 5.**
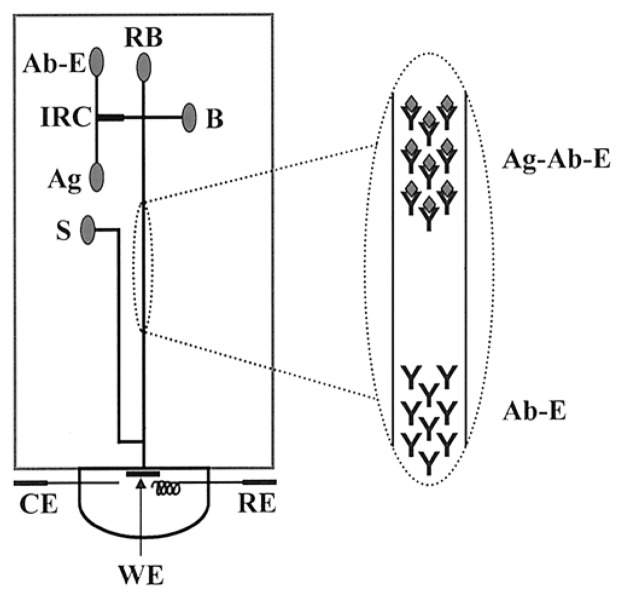
Schematic of the microchip-based electrochemical enzyme immunoassay. From Wang *et al.*, *Anal. Chem.* **2001**, *73*, 5323-5327. Reprinted with permission from ACS.

Microfluidics, a branch of MEMS that handles fluid flow in microchannels, offers many advantages to point-of-care protein detection. The most obvious one is reduction in sample and reagent volumes. Another attractive capability is the fully automated fluid control. For example, the advanced microfluidics of the i-STAT analyzer automates the washing of excess enzyme conjugate and enables the amperometric detection of cTnI in a sandwich ELISA format in just a few minutes. Besides washing function, microfluidics permits rapid separation of free antibody and antibody-antigen complex in solution phase by microchip capillary electrophoresis. Wang et al. developed a microfluidic device for performing electrochemical enzyme immunoassays (Figure 5) [78]. The assay procedure started with the mixing of an antibody–enzyme conjugate (Ab-E) and antigen (Ag) in an immunoreaction chamber (IRC), followed by electrophoretic separation of the free (Ab-E) and antigen-bound (Ag-Ab-E) antibodies. The substrate (S) of the enzyme was introduced close to the end of the separation channel and the product was measured amperometrically with an end-column three-electrode system (WE: screen-printed carbon working electrode; CE: counter electrode; RE: reference electrode).

**Figure 6.**
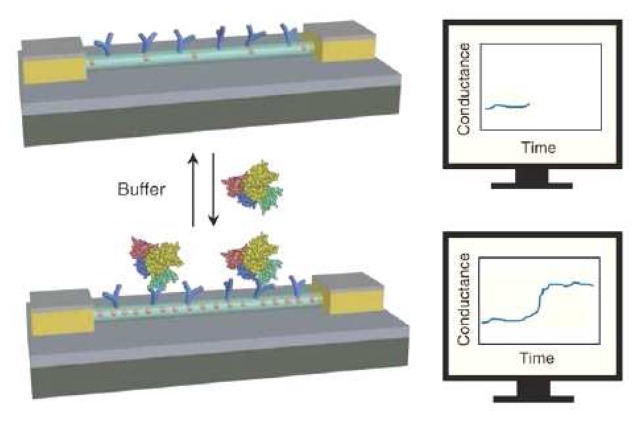
Schematic of the silicon nanowire sensor for label-free and real-time protein detection. From Patolsky *et al.*, *MRS Bulletin* **2007**, *32*, 142-148. Reprinted with permission from MRS.

At present, nanobiosensors hold great promise for protein detection owing to their ultra-high sensitivity and multiplexity, as well as label-free and real-time measurements. In 2005, Lieber's group fabricated a silicon-nanowire field-effect device for multiplexed electrical detection of cancer markers [79]. The conductance of the antibody-functionalized silicon nanowire was strongly dependent on its surface charges. As shown in Figure 6, the conductance of a p-type (boron-doped) silicon nanowire increased instantaneously upon the specific binding of a negatively charged target protein. Another important type of protein nanobiosensors is carbon nanotube field-effect transistor [80, 81]. For the mobile charges of the silicon nanowire and carbon nanotube field effect transistor to pick up the surplus surface charges, the recognition event must occur within the electrical double layer (i.e., Debye length, ∼3 nm in a 10 mM ionic strength). Because of this, aptamer (size of ∼2 nm) has been shown to be a better recognition molecule than antibody (size of ∼10 nm) in a carbon nanotube field-effect transistor [82].

## 4. DNA Sensors

DNA assays are currently standard methods for the identification of a large number of genetic and infectious diseases in clinical laboratories. With the significant researches on electrochemical DNA sensors over the past 15 years, home-use devices would be realized soon. The basic components of an electrochemical DNA sensor include the immobilization of an oligonucleotide probe onto an electrode, hybridization of a complementary target sequence, and transduction of the hybridization event. Numerous transduction schemes have been developed and they can be classified as indicator-based or indicator-free approach. The first electrochemical DNA sensor was reported by Millan and Mikkelsen, the transduction of which was based on an electroactive indicator of tris(2,2′-bipyridyl)cobalt(III), Co(bpy)_3_^3+^ [83]. This redox indicator binds more strongly to double-stranded DNA (dsDNA) than to single-stranded DNA (ssDNA) in an intercalative mode, thus the voltammetric current signal increases if the sample contains the complementary target sequence. In addition to Co(bpy)_3_^3+^, other metal complexes (e.g., tri(1,10-phenanthroline)cobalt(III), Co(phen)_3_^3+^[84]) and organic molecules (e.g., Hoechst 33258 [85], daunomycin [86, 87], methylene blue [88, 89], and 2,6-disulfonic acid anthraquinone [90]) have been employed as hybridization indicators. Of great significance, Barton and coworkers have studied the charge transport between the redox intercalator (especially daunomycin and methylene blue) and a gold electrode through DNA [91]. The experiments involved the immobilization of a short duplex (15 bp) via gold–thiol linkage (the 5′ end of one strand was labeled with a thiol group), forming a densely packed DNA film on the gold surface. Subsequent to a washing step, the DNA-modified electrode was immersed in an intercalator solution and studied by cyclic voltammetric measurement. The compact structure resulted in the binding of the intercalator close to the top of the film (i.e., farthest from the electrode surface), so charge transport was achieved through DNA. Strikingly, the presence of a single-base mismatch in the duplex caused a huge decrease in the current signal. It should be pointed out that these redox intercalators do not necessarily bind preferentially to dsDNA. For example, methylene blue has been shown to interact with exposed guanine base specifically [92], therefore, the current signal of the probe-target electrode was lower than that of the probe-only electrode [93]. The assay sensitivity of these redox intercalators depends strongly on their binding properties (e.g., binding constant and dissociation rate constant). Synthetic threading intercalator (naphthalene diimide derivative) with better superior binding properties has been functionalized with ferrocene redox marker, giving rise to a highly sensitive electrochemical hybridization indicator [94].

Non-DNA binding soluble redox species could also be used for transducing the hybridization event. Thorp and co-workers demonstrated that tris(2,2′-bipyridyl)ruthenium(III), Ru(bpy)_3_^3+^, catalyzed the oxidation of guanine [95]. In terms of the construction of the DNA sensor, guanine bases in the probe were replaced by inosine bases, which could base-pair with cytosine but much less reactive toward Ru(bpy)_3_^3+^. This was significant in that the probe-only surface had a low background signal and thus the hybridization of the guanine-containing complementary sequence could lead to a larger change in signal (i.e., higher signal-to-background ratio). In other words, higher detection sensitivity could be obtained. Another approach was based on the ion-channel sensor technique, which took advantage of the electrostatic interactions between a soluble redox marker and DNA-modified electrode surface [96, 97]. In one configuration, a gold electrode was modified with a mixed monolayer of peptide nucleic acid (PNA) and 6-mercapto-1-hexanol. PNA contains a neutral *N*-(2-aminoethyl)glycine backbone in contrast to DNA's negatively charged sugar-phosphate backbone. Due to the lack of electrostatic repulsion, PNA has a higher binding affinity with its complementary DNA sequence than its DNA counterpart. A negatively charged redox marker, Fe(CN)_6_^3−/4−^, could access the electrode surface in the absence of the target sequence, while the hybridization resulted in electrostatic repulsion and the voltammetric current was significantly reduced.

To enhance the sensitivity of the repulsion-based DNA sensors, Willner and co-workers developed a novel amplification strategy using functionalized liposomes [98]. Their protocol commenced with the immobilization of an oligonucleotide capture probe onto a gold electrode. The capture probe was designed to hybridize with one part of the complementary target sequence. After the target hybridization, the other part of the target was allowed to hybridize in a sandwich format with a detection probe linked to a negatively charged liposome. The interfacial electron transfer resistance for this giant negatively charged interface with the redox marker of Fe(CN)_6_^3−/4−^ was measured by Faradaic impedance spectroscopy. Apart from liposomes, the same group labeled the detection probe with horseradish peroxidase via biotin–avidin linkage that catalyzed the oxidation of a soluble compound (4-chloro-1-naphthol) to form an insoluble product covering the electrode surface [99]. This created a very high barrier for interfacial electron transfer and was probed by Faradaic impedance spectroscopy with Fe(CN)_6_^3−/4−^ redox marker. The electrical wiring of redox oxidase within a redox hydrogel, as in glucose sensor by Heller's group, has been extended to DNA sensing [100]. In this case, a capture probe was immobilized within the redox hydrogel. Upon the hybridization of the target and horseradish peroxidase-labeled detection probe, the electrical contact between the horseradish peroxidase and redox hydrogel was established. The amount of target sequence present in the sample was proportional to the hydrogen peroxide electroreduction current obtained.

Nanomaterials, particularly metal and semiconductor nanoparticles, have received a great deal of attention as hybridization indicators. In 2002, Mirkin and co-workers reported a novel electrical DNA detection scheme using oligonucleotide-modified gold nanoparticle [101]. The assay involved a pair of microelectrodes (patterned on a SiO_2_-coated silicon wafer by photolithography) with a 20-mm gap in between. A capture probe was immobilized onto the gap, followed by the hybridization of the target and oligonucleotide-modified gold nanoparticle in a sandwich format. At this point, the gold nanoparticles were brought close to, but separated from, one another. A silver enhancement step was then carried out to deposit silver metal onto the gold nanoparticle preferentially, thereby bridging the gap and the resistance across the pair of electrodes dropped dramatically. Electrochemical detection of specific DNA sequences with gold nanoparticle probes on conventional electrodes has also been achieved, which involved the direct oxidation of the gold nanoparticles [102] or silver metal after an enhancement step (either chemical [103, 104] or electrocatalytic deposition [105]). One drawback of these gold nanoparticle probes is that they can only be used for single target detection, unless an array of electrodes are employed. On the other hand, semiconductor nanocrystals allow simultaneous detection of multiple targets based on their well-defined and diverse redox potentials. Wang's group demonstrated the detection of three targets in a single tube and voltammetric run using zinc sulfide, cadmium sulfide, and lead sulfide nanoparticles [106]. Furthermore, using four nucleoside monophosphate–semiconductor nanoparticle conjugates, they distinguished all the eight possible single-base mismatches in a single run [107]. These two studies made use of magnetic beads as the biorecognition support. After stringent washing to remove excess nanoparticle labels, the magnetic bead-bound labels were dissolved by an acidic treatment and finally quantified by anodic stripping voltammetry using a glassy carbon disc working electrode.

**Figure 7.**
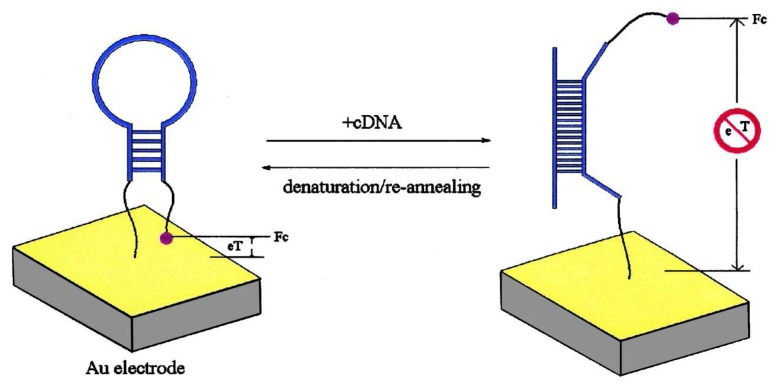
Schematic diagram illustrating the mechanism of the molecular beacon type electrochemical DNA sensor. From Fan *et al.*, *Proc. Natl, Acad. Sci. USA*, **2003**, *100*, 9134-9137. Copyright (2003) National Academy of Sciences, U.S.A.

Almost all of the above mentioned indicator-based approaches require thorough washing steps to remove unbound noncomplementary sequences and indicators. One wash-free indicator-based approach was developed by Motorola's Clinical Micro Sensors Division (eSensor™) [108]. A gold electrode was modified with a self-assembled monolayer (SAM) of capture probe, oligophenylethynyl (as molecular wire), and polyethylene glycol (as insulator). The sample was mixed with a detection (or signaling) probe containing ferrocene-modified nucleotides. The presence of the target sequence held the signaling probe in close contact with the molecular wire, thereby facilitating electron transfer between the ferrocene redox markers and the gold electrode. The unhybridized probes were effectively blocked by the polyethylene glycol insulator. Another wash-free format that eliminated the need of a signaling probe and molecular wire was developed by Plaxco and co-workers, which took advantage of the distance-dependent charge transfer property resulting from the conformational change of a molecular beacon type capture probe labeled with a ferrocene marker [109]. The molecular beacon probe had a thiol at its 3′ end for immobilization onto a gold electrode and a ferrocene redox marker at its 5′ end. The probe had an initial stem–loop structure that kept the ferrocene marker close to the electrode surface for efficient electron transfer (left panel of Figure 7). When a complementary sequence hybridized with the probe, the stem–loop structure was straightened out and thus the electron transfer was greatly reduced (right panel of Figure 7). Modifications of the capture probe design switched the architecture from signal-off to signal-on mode [110, 111].

**Figure 8.**
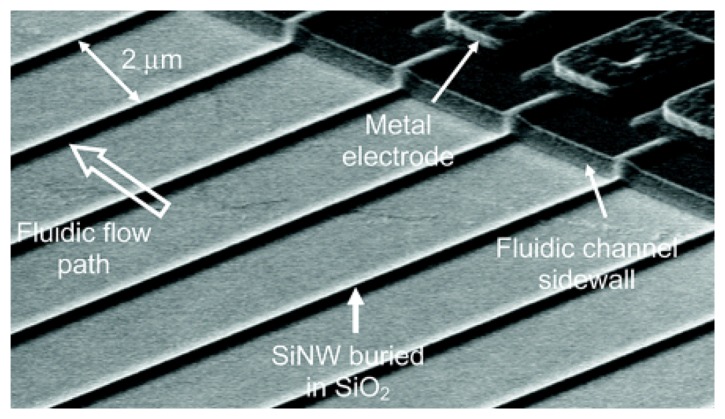
Scanning electron microscope photograph of a silicon nanowire array fabricated by CMOS compatible technology. From Gao *et al.*, *Anal. Chem.* **2007**, *79*, 3291-3297. Reprinted with permission from ACS.

In fact, the hybridization event can be detected without the use of an indicator. One indicator-free approach relies on the intrinsic electroactivity of DNA bases [112-114]. The most redox active nitrogenous base in DNA is guanine, which has an oxidation peak at ∼+1.0 V with carbon paste electrode [112-114] versus Ag/AgCl reference electrode and at +0.73 V with gold electrode [115]. In these cases, the guanine bases in the capture probes were replaced by inosine. Another approach takes advantage of the changes in interfacial electrical properties (e.g., capacitance [116] and non-Faradaic impedance [117-119]) upon the hybridization of negatively charged target DNA on electrode surfaces. One facile method that does not involve laborious and time consuming chemical modification of the electrode surface is based on the doping of capture probe within electropolymerized polypyrrole [120-122]. This is particularly useful for multiplexed detection with an array of closely-spaced microfabricated electrodes. With advanced microelectronics fabrication techniques, silicon field-effect transistors have been utilized for electrical transduction of the hybridization event [123-126]. Further miniaturization of the field-effect DNA sensors with ultra-high sensitivity has been demonstrated by Lieber's group using silicon nanowires [127]. The main challenge faced by these silicon nanowire sensors is the fabrication, which involved nanowire synthesis by chemical vapor deposition and electrical wiring by electron-beam lithography. These processes do not favor mass production that is essential for successful commercialization. In view of this, very recently, Gao et al. reported the fabrication of silicon nanowire arrays using complementary metal-oxide semiconductor compatible technology [128]. The mass production capability is conducive to the production of low-cost devices for routine diagnostics. As shown in Figure 8, an array of silicon nanowire sensors was constructed, which could allow multiple analytes to be detected simultaneously.

**Figure 9.**
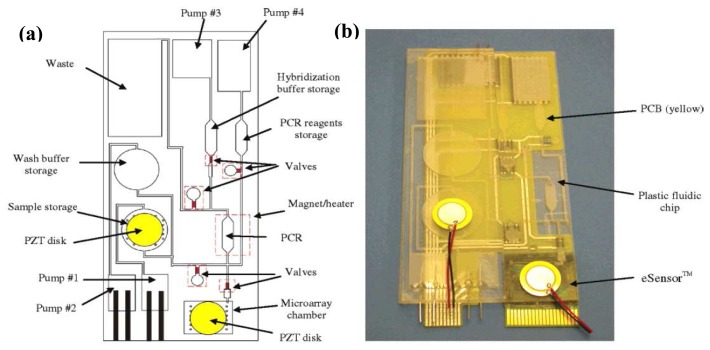
(a) Schematic and (b) photograph of the self-contained, fully integrated DNA biochip developed by Motorola. From Liu *et al.*, *Anal. Chem.* **2004**, *76*, 1824-1831. Reprinted with permission from ACS.

It must be emphasized that most of the reported electrochemical/electrical DNA sensors use synthetic short oligonucleotide as the model targets. Problems could arise when dealing with real samples as a result of the huge steric hindrance encountered by very large targets (thousand to several hundred thousand base pairs). Some studies addressed this issue using polymerase chain reaction (PCR) amplicons as targets (typically several hundred base pairs). Moreover, coupling electrochemical DNA sensors to PCR could increase the assay sensitivity tremendously. A complete DNA analysis usually involves multiple steps of DNA extraction and isolation, target DNA amplification by PCR, and finally PCR product detection. In this regard, MEMS and microfluidics technologies enable all these to be carried out in a handheld instrument that can be operated by an untrained person. In 2004, Motorola's Microfluidics Laboratory developed a fully integrated plastic biochip that contained microfluidic mixers (piezoelectric disk made of lead zirconate titanate, PZT disk), valves, pumps, channels, chambers, heaters, and DNA sensors (microarray chamber) for performing sample preparation, PCR, and electrochemical sequence-specific PCR product detection [129] (Figure 9). Indeed, the electrochemical DNA sensor used was the wash-free indicator-based eSensor™ also developed by Motorola. The entire assay took about three hours (30 min of sample preparation, 90 min of amplification, and 60 min of detection). In an attempt to shorten the assay time, a special technique termed as electrochemical real-time PCR was developed by Hsing et al. that featured simultaneous PCR amplification and electrochemical detection in a silicon-glass microdevice [130]. The silicon chip contained a PCR microchamber as well as integrated thin-film platinum heater and temperature sensor for precise and fast thermal control. The glass chip, which sealed the microchamber, had four indium tin oxide working electrodes together with platinum pseudoreference and counter electrodes patterned on its surface for electrochemical measurements. The working electrodes were functionalized with a capture probe that, at the annealing step of PCR, hybridized with the PCR amplicon. The conventional PCR recipe was slightly modified by substituting ferrocene-labeled deoxyuridine triphosphate for deoxythymine triphosphate. At the extension step, the capture probe was elongated by the polymerase with the incorporation of ferrocene labels. A progressive accumulation of the redox marker onto the electrode surface in response to the target amplicon generation was observed.

## 5. Conclusions

With about 45 years of development, biosensors have had huge commercial value, mainly from glucose meters. The technologies for the electrochemical detection of other key metabolites, proteins, and DNAs are quite mature. For all these biosensors to reach everybody's hand, key issues remain to be addressed are their cost, simplicity, and speed. Continuous researches have to be carried out in all the building blocks of biosensors, which include transducers, recognition molecules, immobilization strategies, as well as transduction mechanisms. Furthermore, efforts have to be made to take full advantages of nanotechnology and MEMS/microfluidics technology for label-free, real-time, highly sensitive, and multiplexed biomolecular detection in fully automated fashion. These technologies would enable routine health check at home, thereby detecting any abnormalities at an early stage. Last but not least, the results from these new generation biosensors and biochips must be well-validated against existing clinical standards in order to get market acceptance as wide as that of the glucose meters.

## Figures and Tables

**Table 1.** Comparisons between four commercial fingerprick-type glucose meters.

	**Abbott FreeStyle Lite**	**Bayer Contour**	**LifeScan OneTouch Ultra2**	**Roche Accu-Chek Aviva**
Sample Size	0.3 μL	0.6 μL	1 μL	0.6 μL
Test Range	1.1 – 27.8 mM	0.6 – 33.3 mM	1.1 – 33.3 mM	0.6 – 33.3 mM
Test Time	5 s	5 s	5 s	5 s
Alternative Site Testing	Hand, forearm, upper arm, thigh, or calf	Palm or forearm	Palm or forearm	Palm, forearm, upper arm, thigh, or calf
Memory	400 results	480 results	500 results	500 results
Special Feature	No coding required	No coding required, 7, 14, and 30-day averages	Link after meal results with food and portion choices	7, 14, and 30-day averages

**Table 2.** Comparisons between four commercial lactate meters.

	**ApexBio The Edge**	**Arkray Lactate Pro**	**EKF Diagnostic Lactate Scout**	**Nova Biomedical Lactate Plus**
Sample Size	3 μL	5 μL	0.5 μL	0.7 μL
Test Range	1.1 – 22.2 mM	0.8 – 23.3 mM	0.5 – 25.0 mM	0.3 – 25.0 mM
Test Time	45 s	60 s	15 s	13 s
